# Effectiveness of an mHealth-Based School Sodium Reduction Scaling-Up Program in Three Chinese Cities: A Real-World Pre–Post Study Among Adults and Children

**DOI:** 10.3390/nu18142314

**Published:** 2026-07-15

**Authors:** Yang Zhang, Yan Liu, Yinghua Li, Yuan Li, Li Li, Kaige Sun, Tao Mao, Naibo Wang, Gaoqiang Xie, Liuruyu Yu, Feng J. He, Puhong Zhang

**Affiliations:** 1 Zhenjiang Center for Disease Control and Prevention, Zhenjiang 212000, China; 2 Department of Monitoring and Evaluation, Chinese Centre for Health Education, Beijing 100011, China; 3 Food Policy Division, The George Institute for Global Health, Faculty of Medicine, University of New South Wales, Sydney, NSW 2052, Australia; 4 Research and Education Department, Beijing Physical Examination Center, Beijing 100068, China; 5 Department of Health Education, Jiangsu Provincial Center for Disease Control and Prevention, Nanjing 210009, China; 6 Jiangxi Provincial Key Laboratory of Preventive Medicine, School of Public Health, Jiangxi Medical College, Nanchang University, Nanchang 330006, China; 7 Department of Data Management, Clinical Research Institute, Institute of Advanced Clinical Medicine, Peking University, Beijing 100191, China; 8 Key Laboratory of Epidemiology of Major Diseases, Peking University, Ministry of Education, Beijing 100191, China; 9 Wolfson Institute of Population Health, Queen Mary University of London, London EC1M 6BQ, UK

**Keywords:** sodium reduction, mobile health, school-based health education, knowledge–attitude–practice (KAP) theory, urinary sodium

## Abstract

**Objective:** To systematically evaluate the impact of a school-based sodium reduction education program using mobile health (mHealth), known as EduSaltS, on salt-related knowledge, attitudes, practices (KAP) and sodium intake among adults and children during its real-world scaling-up and to examine geographic and population heterogeneity in its effects. **Methods:** A multi-center pragmatic pre–post study design was used. The EduSaltS program was implemented in three cities (Ganzhou in southern China, Zhenjiang in eastern China, and Qinhuangdao in northern China) between April 2022 and July 2024. Ten primary schools were selected from each city. Baseline and follow-up surveys were conducted approximately 12 months apart, aligned with the school calendar. Electronic questionnaires were used to collect baseline and post-intervention data on the primary outcome—salt-related KAP—from adults and children. In Ganzhou and Zhenjiang, 24 h urine collections were conducted to measure urinary sodium excretion. **Results:** A total of 721 adults and 770 children were included in the final analysis. After the intervention, the total KAP scores significantly increased in both adults and children across the three sites: by 8.7–10.0 points in adults and by 11.4–16.7 points in children (all *p* < 0.001). The changes in sodium intake showed significant heterogeneity. A significant decrease in urinary sodium was observed among adults in Ganzhou (−14.0 mmol/24 h, 95% CI: −26.1, −2.0, *p* = 0.022), corresponding to a reduction of 0.82 g in daily salt intake. No significant change was found among adults in Zhenjiang (adjusted difference: −0.7 mmol/24 h, 95% CI: −13.4 to 12.0, *p* = 0.912). In children, after the one-year intervention, urinary sodium excretion showed a significant increase from baseline to follow-up in both cities (Ganzhou: +12.9 mmol/24 h, *p* = 0.018; Zhenjiang: +14.0 mmol/24 h, *p* = 0.0002). **Conclusions:** The pre–post evaluation showed that the EduSaltS program was associated with significant improvements in salt-related knowledge across regions. Its effectiveness in reducing sodium intake, however, varied substantially by population and geographic location. The observed increase in children’s urinary sodium excretion may partly reflect normal childhood growth and the limitations of a pre–post design without a control group. Pragmatic randomized controlled trials are warranted to verify the effectiveness of such interventions in reducing sodium intake and blood pressure across diverse populations and settings.

## 1. Introduction

Cardiovascular disease (CVD) is the primary cause of death and disability worldwide, imposing a substantial health burden and economic loss [1]. Hypertension is the most important and modifiable risk factor for CVD, and its prevalence continues to rise [2]. Among the many causes of hypertension, excessive salt (sodium) intake is a key, modifiable dietary risk factor. Strong evidence indicates that high salt intake not only directly elevates blood pressure but also independently increases the risk of stroke, coronary heart disease, and chronic kidney disease [3,4].

The World Health Organization (WHO) has prioritized population-level salt reduction as a highly cost-effective public health strategy. Its goal is to cut global average salt intake by 30% by 2025 [5]. However, despite salt reduction initiatives having been launched in many countries and regions worldwide, overall progress remains slow and uneven [6]. Long-established salty eating habits and taste preferences are hard to break. At the social level, traditional home cooking practices are deeply rooted. And at the environmental level, widespread “hidden salt” in processed foods and restaurant meals makes it difficult for consumers to even know, let alone control, their total sodium intake [7,8]. We urgently need new intervention models that are innovative, scalable, and able to break through these barriers.

Against this background, two major trends are opening up new opportunities for public health interventions. First, schools—as institutional settings that reach nearly all children and adolescents—are widely recognized as an ideal platform for health promotion and for building lifelong healthy habits [9]. Through schools, we can not only reach students directly but also influence their families indirectly, especially the parents who shop for groceries and cook. Second, the rapid growth of mobile health (mHealth) technologies and the widespread availability of smartphones have provided unprecedented tools for delivering health information and behavioral guidance at scale, at low cost, and in a standardized way [10]. With high accessibility, strong interactivity, and personalization, mHealth interventions help overcome the limitations of traditional in-person health education in terms of coverage and long-term sustainability.

Our team previously designed the School-EduSalt and AppSalt programs, which were proven effective in reducing sodium intake among children and their families through randomized controlled trials [11,12]. To further examine its scalability and effect in real-world implementation, we conducted a scaling-up study, named EduSaltS, which rolled-out the mHealth-based school-student-family health education among the primary schools in three cities in China from 2022 to 2024. EduSaltS aimed to systematically improve salt-related knowledge, attitudes, and practices (KAP) among students and their families through a one-academic-year intervention comprising online courses, offline activities, and supportive environmental changes. Although an embedded RCT conducted in Ganzhou (one of the three scaling-up cities) and a recent study pooling three RCTs have demonstrated the effectiveness of mHealth-based school health education for sodium reduction, both focused primarily on RCT-based effects, with no in-depth analysis of effect heterogeneity across different sub-populations [13,14].

We hypothesized that the EduSaltS program would improve salt-related KAP and reduce sodium intake, with effects varying by geographic location and population. To test these hypotheses, this study aims to conduct an in-depth analysis of data from EduSaltS program with diverse socioeconomic and dietary culture backgrounds, with the following core objectives: (1) to evaluate the effectiveness of the EduSaltS program in improving self-reported salt-related KAP among adults and children under real-world conditions (primary outcome); (2) to assess the program’s impact on sodium intake measured by 24 h urinary sodium excretion in both adults and children; and (3) to examine the heterogeneity of intervention effects across different cities (Ganzhou in southern China, Zhenjiang in eastern China, and Qinhuangdao in northern China) and populations (adults vs. children) and to explore the underlying reasons based on the health ecological model [15].

## 2. Materials and Methods

### 2.1. Study Design

The EduSaltS program is a school-based sodium reduction intervention that uses a WeChat mini-program (Tencent, Shenzhen, China) to deliver health education, complemented by school-based theme activities and supportive environmental changes. During its scale-up from 2021 to 2024, all of the 70,000 grade 3 students and their families from 308 primary schools were engaged in the innovative education program for a school year. To assess overall and city-specific effectiveness, we surveyed the same randomly sampled participants at baseline and after the scaling-up intervention, and here report the results of this quasi-experimental study.

### 2.2. Participants

In each city, a cluster random sampling method was used to select evaluation schools from the pool of schools that were implementing the EduSaltS intervention: 10 out of 108 schools in Zhenjiang, and 10 out of 100 schools in each of Ganzhou and Qinhuangdao. Thus, a total of 30 schools (10 per city) were selected for outcome evaluation. For each school, 26 grade 3 students and one primary caregiver living with them were recruited. Inclusion criteria were: (1) enrollment in the third grade of a participating school with no intention to transfer out during the following academic year; (2) age 18–75 years for adult participants; and (3) voluntary participation, signed informed consent, and commitment to complete follow-up. To reduce bias from gender imbalance, one adult caregiver who lived and ate with the child was selected from each family based on the following rules: for a boy, the mother or maternal grandmother living with him was preferred; if both were willing, the one who could more easily participate was chosen; if neither could participate, the father or paternal grandfather living with him was selected. For a girl, the father or paternal grandfather was preferred; if both were willing, the more convenient one was chosen; if neither could participate, the mother or maternal grandmother living with her was selected. Exclusion criteria were: (1) renal insufficiency; (2) explicitly stating inability to complete follow-up; and (3) for cities where urine collection was required (i.e., Ganzhou and Zhenjiang, but not Qinhuangdao), being unable or unwilling to collect 24 h urine.

### 2.3. Intervention

The intervention was a school-based sodium reduction education program using mobile health (mHealth). Its core components included: weekly cartoon-style sodium reduction lessons delivered through the “Healthy Salt Classroom” WeChat mini-program, at least four school-based sodium reduction theme activities per semester, and supportive environmental changes on campus. All the grade 3 students and their families in the 308 schools received the same intervention; no parallel control group was included. The program design and implementation procedures have been described in detail in our previous publications [13].

The study period partially overlapped with the COVID-19 pandemic in China. During the 2022 wave, when schools were temporarily closed, the intervention continued online as scheduled. Students and their families accessed the health education lessons via the WeChat mini-program from home, and class teachers sent weekly reminders through the online group to encourage participation and ensure course completion. This online-based delivery mode, which had been designed as the core component of the intervention, ensured that the program could continue without interruption during periods of school closure. No major disruptions to intervention delivery or data collection were reported by the participating schools, and adherence remained satisfactory.

### 2.4. Outcome Measures

Trained field staff from local CDCs and township health centers conducted assessments at baseline and at the 12-month follow-up. The assessment protocol covered four domains: (a) sociodemographic characteristics and health behaviors (collected from adults); (b) standardized measurements of height, weight, and blood pressure; (c) self-administered electronic KAP questionnaires (child and adult versions); and (d) 24 h urine collection. The specific methods for each outcome are detailed below.

#### 2.4.1. Primary Outcome

The primary outcome was the change in salt-related knowledge, attitudes, and practices (KAP) from baseline to the one-year follow-up. Two separate electronic questionnaires—one for children and one for adults—were developed and validated for this study.

Each questionnaire consisted of three subscales: knowledge (e.g., sources of dietary sodium, recommended daily limits, health risks of excess sodium), attitudes (e.g., willingness to reduce salt, perceived benefits of a low-sodium diet), and practices (e.g., frequency of adding salt during cooking, use of salty condiments, reading nutrition labels).

For each subscale, individual items were scored on a 0–10 scale. The subscale score was the average of the item scores, ranging from 0 to 10. The total KAP score was the sum of the three subscale scores, giving a range of 0 to 30. Higher total scores represented better salt-related knowledge, more favourable attitudes, and healthier practices.

#### 2.4.2. Secondary Outcomes

Sodium intake was measured using 24 h urinary sodium excretion, which is the gold-standard method for estimating dietary sodium consumption. Participants collected all urine over a complete 24 h period. After recording total urine volume, an aliquot was sent to a central laboratory for sodium concentration analysis. The 24 h urinary sodium excretion (in mmol/24 h) was calculated as sodium concentration (mmol/L) multiplied by total urine volume (L). Daily salt intake (g/day) was derived as urinary sodium excretion (g/day) multiplied by 2.54. Completeness of urine collections was verified by comparing urinary creatinine excretion with age- and sex-specific reference ranges; collections outside the expected range were excluded. This method is considered the gold standard for assessing sodium intake [16].

Blood pressure (BP) was measured at baseline and follow-up using an automated oscillometric device (Omron Healthcare Co., Ltd., Kyoto, Japan) on the right arm after a 10-min seated rest. Three readings were taken with at least one minute between them, and the average of the last two measurements was used for analysis. Systolic and diastolic BP values are reported.

### 2.5. Statistical Analysis

SAS version 9.4 (SAS Institute Inc., Cary, NC, USA) was used for all analyses. Continuous variables were described as mean ± standard deviation, and categorical variables as frequency (percentage). Paired *t*-tests were used to compare changes in KAP scores and urinary sodium excretion before and after the intervention within each group. Mixed linear models (adjusting for school clustering effects) were applied to estimate the overall intervention effects. Analysis of covariance (ANCOVA) was used to compare post-intervention KAP scores across the three cities, adjusting for baseline scores and demographic covariates. A two-tailed *p* value < 0.05 was considered statistically significant.

## 3. Results

### 3.1. Baseline Characteristics of Study Participants

**Figure 1:**
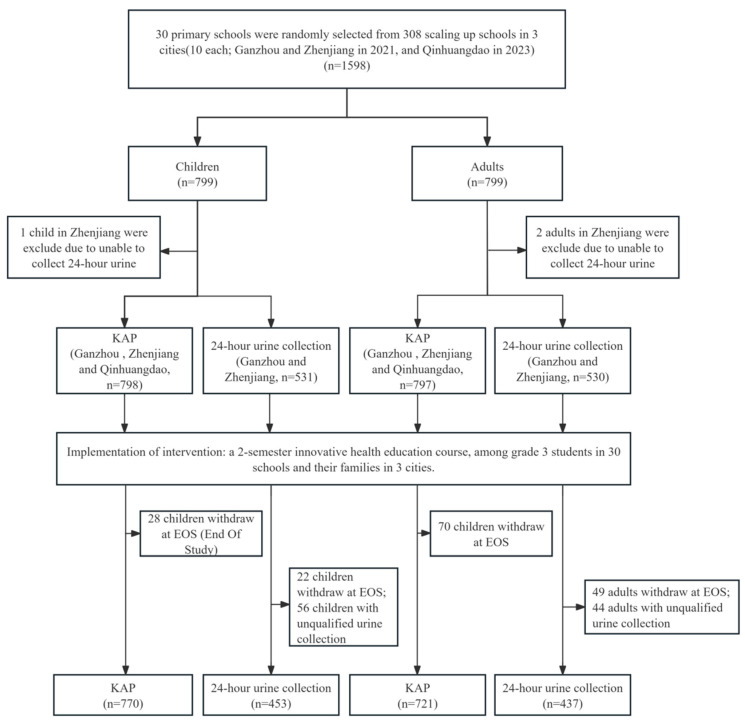
Flow chart of participant selection in EduSaltS, showing enrollment, follow-up, and final analytical samples.

A total of 799 children and 799 adults were enrolled at baseline. After one academic year of intervention, losses to follow-up and invalid urine samples resulted in a final KAP sample of 770 children and 721 adults. The 24 h urinary sodium analysis included 453 children and 437 adults from Ganzhou and Zhenjiang (no urine samples were collected in Qinhuangdao, as the implementation there was delayed by one year due to the COVID-19 pandemic and could not be synchronized with the other two cities for the 24 h urine collection). The participant flow chart is presented in Figure 1.

The mean age was 8.6 ± 0.5 years for children and 39.1 ± 9.6 years for adults. Baseline characteristics (e.g., sex, age, BMI) were generally balanced across the three cities. The overall follow-up rate exceeded 90%. Detailed baseline characteristics and comparisons between different datasets are presented in Table 1.

### 3.2. Impact of the Intervention on KAP Scores

Intervention adherence was assessed using process data from the broader EduSaltS implementation [13]. The program achieved 100% school participation, a 98% family registration rate, and an average course completion rate of 83.5% across all cloud lessons, with stable course completion rates ranging from 79.4% to 93.4% over the one-year intervention period. School-based activity participation showed a median of 3.0 activities per class (IQR: 1.0–7.0) and 9.0 activities per school (IQR: 4.0–12.0). These data suggest that engagement with the intervention was generally satisfactory.

After one year of intervention, total KAP scores and subdomain scores improved significantly in both adults and children across all three cities (all *p* < 0.0001). Among children, the largest increase in KAP scores was observed in Qinhuangdao (+16.7 points), which was significantly higher than in Ganzhou (+11.4 points) and Zhenjiang (+14.6 points) (*p* for subgroup difference < 0.001). Among adults, the increases were similar across the three cities (ranging from 8.7 to 10.0 points), with no significant geographic differences. Detailed changes are presented in Table 2.

### 3.3. Impact of the Intervention on Urinary Sodium Excretion

In Ganzhou and Zhenjiang, the patterns of urinary sodium change differed markedly. Among adults, a significant decrease was observed only in Ganzhou (155.9 ± 65.5 vs. 141.9 ± 59.6 mmol/24 h; adjusted difference: −14.0 mmol/24 h, *p* = 0.0224), while no significant change was found in Zhenjiang (148.8 ± 63.4 vs. 148.1 ± 75.6 mmol/24 h; adjusted difference: −0.7 mmol/24 h, *p* = 0.9122). In children, urinary sodium excretion showed a significant increase from baseline to follow-up in both cities (Ganzhou: +12.9 mmol/24 h, *p* = 0.018; Zhenjiang: +14.0 mmol/24 h, *p* = 0.0002). Detailed data are presented in Table 3.

### 3.4. Subgroup Analysis

To further explore the heterogeneity of intervention effects, subgroup analyses of changes in adult KAP scores were performed by sex, education level, and hypertension status (Table 4). The results showed that KAP scores improved significantly from baseline across all subgroups (all *p* < 0.001). In terms of magnitude, the improvement was larger in females (adjusted difference: 10.7 points, 95% CI: 9.0–12.4) than in males (7.4 points, 95% CI: 5.0–9.9). Participants with secondary education (junior/senior high school) showed the greatest improvement (11.1 points, 95% CI: 9.1–13.0), compared with those with primary education or below (7.4 points) and those with college education or above (7.0 points). Hypertension status had no apparent impact on KAP improvement (both around 9.5 points). After the intervention, adult urinary sodium showed no statistically significant differences by city, sex, age, education level, per capita household income, or relationship with the child (Table 5). Subgroup analyses in children showed that KAP scores improved significantly across different sex and age groups, but the general trend of increased urinary sodium excretion was consistent across subgroups (Table 4 and Table 5).

## 4. Discussion

This large-scale pragmatic study conducted in three representative Chinese cities revealed both the opportunities and challenges faced by a comprehensive sodium reduction health education program in complex real-world settings. Our findings can be interpreted at multiple levels within the framework of the Health Ecological Model, which emphasizes that health behaviors are the result of interactions among multiple factors, including individual characteristics, interpersonal networks, community environments, and policies [17].

### 4.1. Universality of Cognitive Improvement: Core Strengths of the Program and the Empowering Role of mHealth

The most consistent finding of this study was the significant improvement in KAP scores achieved by the EduSaltS program. This result aligns with recent systematic reviews on the effectiveness of digital health interventions, which generally show that mHealth strategies produce small-to-moderate effect sizes that are statistically significant in improving health knowledge and social cognition [18]. The success of the program can be attributed to several key factors. First, the standardized digital curriculum ensured quality control and consistent delivery of intervention content, overcoming the variability in effectiveness often seen in traditional health education due to differences in instructor competence [19]. Second, gamified interactions and real-time feedback made learning more engaging and increased participant involvement, which was essential for sustaining motivation among children and their family members [20]. Finally, the organizational support of the school environment provided the structure and compulsion needed to ensure high participation rates and course completion—an advantage rarely achieved in community-based interventions [21]. Together, these features lay a solid foundation for further nationwide scale-up of the program as a standardized public health tool.

### 4.2. Complexity of Behavioral Change: Interplay of Multi-Level Factors

Our study showed heterogeneity in sodium intake as assessed using the gold-standard method of 24 h urinary sodium excretion. This dissociation between knowledge, attitudes, and practices highlights the substantial challenge of translating health intentions into sustained behavioral change and reveals the complex interplay of multi-level factors underlying behavioral modification [22].

#### 4.2.1. Macro-Level Geographic Context: Food Environment as a Decisive Moderator

Our data clearly show that the food environment at the regional level is a key moderator of intervention effects. The success observed in Ganzhou may reflect a high degree of alignment between the intervention strategy and the primary source of sodium intake in that area. In inland China, salt added during home cooking remains the main contributor to dietary sodium, accounting for 70–80% of total intake [23]. Thus, the core “small hand holding big hand” strategy of the EduSaltS program—which aims to change home cooking behaviors—can precisely target this most important leverage point for sodium reduction. In contrast, the pattern seen in Zhenjiang suggests that environmental barriers may have contributed to the observed differences. In more developed, urbanized areas, dietary patterns are shifting rapidly toward greater consumption of commercial and social foods [24]. Consequently, the proportion of “hidden salt” from restaurants, takeaway meals, and pre-packaged foods has increased substantially [25]. These non-household sources of sodium are not influenced by changes in individual cooking practices, thereby creating a powerful external environmental barrier that can easily offset individual-level efforts [26]. This finding is consistent with the global consensus in sodium reduction: as the primary source of dietary sodium shifts from the household to the marketplace, public health strategies must correspondingly evolve from relying on educating individuals to shaping the food environment [27,28]. From a public health perspective, the observed reduction of 0.82 g/day in salt intake among adults in Ganzhou is modest but clinically meaningful. Previous modelling studies have estimated that a population-wide reduction of 1 g/day in salt intake in China could lower the risk of ischaemic heart disease by about 4% and stroke by about 6%, and could prevent approximately 9 million cardiovascular events by 2030 if sustained [29]. Although the effect size observed in our study was slightly below 1 g/day, the findings suggest that even moderate reductions, if sustained and scaled effectively, could contribute to measurable cardiovascular benefits at the population level.

#### 4.2.2. Micro-Level Individual and Interpersonal Dimensions: Divergent Behavioral Logics of Adults and Children

At the individual level, adults and children follow fundamentally different behavioral logics. The general rise in children’s urinary sodium excretion is one of the findings of this study. Apart from the physiological confounder of rapid growth and development leading to increased total food intake [30], this result more profoundly exposes the structural vulnerability of children in health-related behaviors. According to the “agent-surrogate” theory, children are the “subjects” of health, but their dietary choices are often “surrogated” by adults (parents and schools) [31]. Although children were successfully empowered as health “advocates” through the program, their actual control over their own diet—especially snacks and out-of-school foods—remains very limited [32]. However, children may still exercise independent food choices outside the home, using pocket money to purchase snacks under the influence of peer pressure and food marketing [33]. Their health intentions are easily overwhelmed by powerful commercial forces that aggressively market high-salt, high-sugar foods [34]. Our results suggest that, in the absence of strong environmental regulation, the power of “small hands” is fragile and ineffective against commercial interests.

In contrast, adults have greater behavioral autonomy, but they face their own intrinsic challenges to change. Automaticity of habits, physiological adaptation of taste preferences, and pressure to cope with social situations are all strong internal barriers that sustain a high-salt diet [35,36]. In Ganzhou, the program may have successfully activated adults’ self-regulation capacity and provided sufficient social support (from children) to overcome these barriers. In Zhenjiang, however, the external environmental resistance may have been too large, eroding adults’ self-efficacy and leading them to abandon attempts at behavioral change. We speculate that the attenuated effect in Zhenjiang may be partly explained by contextual factors such as greater reliance on restaurant meals and commercially prepared foods, which are less amenable to individual behavior change [24,25,26]. This time-driven dietary pattern could have exacerbated the challenge of reducing sodium intake, as the “hidden salt” in commercially prepared foods lies largely beyond the reach of individual behavior change. However, we acknowledge that these interpretations remain speculative, as we did not directly measure participants’ food sources or time-use patterns in this study.

### 4.3. Theoretical Integration and Innovation: Toward a New “Empowerment-Environment” Synergistic Model for Sodium Reduction

This study offers important refinements and extensions to classic health behavior theories, such as the knowledge–attitude–practice (KAP) model and the health belief model. It demonstrates that, in today’s highly commercialized food environment, “knowledge” and “attitudes” are necessary but insufficient conditions for behavioral change. We must incorporate “environmental empowerment” as a core component alongside individual empowerment when designing interventions and evaluating their effectiveness [37]. Future behavioral change theories need to better account for how individual agency and environmental structures interact. Drawing on the COM-B (Capability, Opportunity, Motivation—Behavior) model [38] and the intervention ladder framework [39], we propose a new “empowerment-environment” synergistic model for sodium reduction, in which individual empowerment (education, persuasion) enhances capability and motivation—an area where the EduSaltS program has proven effective—while environmental empowerment (environmental redesign, regulation) increases opportunities for behavioral change, which is currently the weak link in the strategy and urgently needs to be strengthened.

### 4.4. Precision Public Health and Policy Implications

Based on the above analysis, we propose the following precise and actionable policy recommendations. First, a geographically stratified strategy should be implemented. A simple assessment tool can be developed to classify cities according to the proportion of sodium from home cooking. In areas with a high proportion of home cooking (the Ganzhou model), programs like EduSaltS should be deployed as a core intervention. In areas with a low proportion (the Zhenjiang model), public health resources should be redirected toward environmental policies such as promoting sodium reduction guidelines in the catering industry, mandating front-of-pack labeling (FOPL), and setting sodium content standards for processed foods. Efforts to reduce sodium in restaurant meals through catering industry reformulation targets are also warranted [40]. Second, population-specific precision interventions should be implemented. For adults, incorporating behavioral change techniques could further enhance program effectiveness [41]. For children, integration with healthy campus legislation and restrictions on high-sodium snacks and marketing would be beneficial [42]. Third, iterative upgrades to mHealth technology should be pursued. Future mHealth platforms could be enhanced with personalized feedback and connections to real-world resources [43].

### 4.5. Strengths and Limitations

The main strengths of this study include its multi-center design, use of urinary sodium biomarkers, high follow-up rate, and concurrent evaluation of both adults and children. Several limitations should be acknowledged. First, the lack of urinary sodium data from Qinhuangdao precluded a comprehensive three-city comparison of sodium intake measured by the most accurate method of 24 h urinary sodium excretion. Second, we were unable to quantitatively measure all potential mediating variables, such as frequency of eating out, food purchasing behaviors, and taste preferences, which limited the depth of our mechanistic analysis. Third, the intervention lasted only one academic year, so its long-term effects and potential “decay effects” remain to be observed. Finally, although we adjusted for multiple covariates, the possibility of unmeasured confounding cannot be ruled out. Additionally, the absence of a parallel control group limits causal attribution, as secular trends or history effects cannot be completely ruled out.

The susceptibility to regression to the mean and secular trends differed across study sites because the evaluation designs were not identical. In Ganzhou, the observed reduction in adult urinary sodium was supported by a concurrent parallel control comparison, which reduces the likelihood that the positive finding was fully explained by regression to the mean, seasonal variation, or broader secular changes. In contrast, the Zhenjiang evaluation relied primarily on a before-and-after comparison without a parallel control group. Therefore, the absence of a significant sodium reduction in Zhenjiang should be interpreted cautiously, as it may have been influenced by external temporal factors, such as seasonal variation in dietary patterns, secular changes in food consumption, or other contextual factors unrelated to the intervention. However, the pragmatic nature of the study prioritizes external validity over internal validity.

## 5. Conclusions

The pre–post evaluation showed that the EduSaltS program was associated with significant improvements in salt-related knowledge across regions. Its effectiveness in reducing sodium intake, however, varied substantially by population and geographic location. The observed increase in children’s urinary sodium excretion cannot be directly attributed to the intervention, as it may partly reflect normal childhood growth and the limitations of a pre–post design without a control group. These findings support shifting from a one-size-fits-all health education approach toward a precision public health paradigm that strengthens environmental empowerment alongside individual empowerment.

## Figures and Tables

**Table 1:** Baseline demographic characteristics of participants in the three cities.

Variables	Children (*n* = 770)	Adults (*n* = 721)
City, *n* (%)		
Zhenjiang	256 (33.2)	236 (32.7)
Ganzhou	253 (32.9)	244 (33.8)
Qinhuangdao	261 (33.9)	241 (33.4)
Gender, *n* (%)		
Boys	404 (52.5)	281 (39.0)
Girls	366 (47.5)	440 (61.0)
Age (year), mean ± SD	8.6 ± 0.5	39.1 ± 9.6
Relationship with children, *n* (%)	-	
Parents	-	617 (85.6)
Grandparents	-	97 (13.5)
Education, *n* (%)	-	
Primary education or less	-	82 (11.4)
Secondary/High school	-	420 (58.3)
College education or above	-	219 (30.4)
Hypertension, *n* (%)	-	126 (17.5)

**Table 2:** Changes in KAP Total Scores Before and After Intervention.

Variable	Baseline (Mean ± SD)	Follow-Up (Mean ± SD)	Adjusted Diff (95% CI), *p*-Value	*p* Value for Heterogeneity Across Subgroups
Adults				0.619
Ganzhou	66.7 ± 15.2	76.3 ± 13.0	9.6 (7.1 to 12.1), <0.001	
Zhenjiang	64.8 ± 14.2	74.8 ± 13.0	10.0 (7.7 to 12.3), <0.001	
Qinhuangdao	65.7 ± 15.6	74.3 ± 15.8	8.7 (5.9 to 11.5), <0.001	
Children				<0.001
Ganzhou	62.5 ± 14.0	73.9 ± 12.7	11.4 (9.3 to 13.5), <0.001	
Zhenjiang	50.3 ± 10.7	64.9 ± 12.0	14.6 (12.7 to 16.5), <0.001	
Qinhuangdao	54.7 ± 12.6	71.4 ± 12.8	16.7 (14.6 to 18.7), <0.001	

Adjusted for cluster (city/school), sex, age, education, and household income.

**Table 3:** Changes in 24 h Urinary Sodium Excretion Before and After Intervention. (Ganzhou & Zhenjiang Only).

Variable	Baseline (mmol/24 h)	Follow-Up (mmol/24 h)	Adjusted Diff (95% CI), *p*-Value	*p* Value for Heterogeneity Across Subgroups
Adults				0.052
Ganzhou	155.9 ± 65.5	141.9 ± 59.6	−14.0 (−26.1 to −2.0), 0.022	
Zhenjiang	148.8 ± 63.4	148.1 ± 75.6	−0.7 (−13.4 to 12.0), 0.912	
Children				0.851
Ganzhou	90.9 ± 39.1	103.8 ± 72.3	+12.9 (2.2 to 23.5), 0.018	
Zhenjiang	87.4 ± 36.4	101.5 ± 43.0	+14.0 (6.7 to 21.4), <0.001	

Adjusted for cluster (city/school), sex, age, and body weight.

**Table 4:** Subgroup Analysis of Changes in KAP Scores by Key Characteristics.

Variable	Baseline (Mean ± SD)	Follow-Up (Mean ± SD)	Adjusted Diff (95% CI), *p*-Value	*p* Value for Heterogeneity Across Subgroups
Adults				
Gender, n (%)				0.005
Male	62.3 ± 15.5	69.7 ± 15.2	7.4 (5.0 to 9.9), <0.001	
Female	67.9 ± 14.3	78.5 ± 12.0	10.7 (9.0 to 12.4), <0.001	
Education, n (%)				0.002
Primary education or less	61.9 ± 15.6	69.3 ± 14.5	7.4 (3.0 to 11.8), 0.001	
Secondary education or High school education	64.7 ± 15.4	75.8 ± 14.1	11.1 (9.1 to 13.0), <0.001	
College education or above	69.0 ± 13.3	76.0 ± 13.1	7.0 (4.7 to 9.3), <0.001	
Hypertension, n (%)				0.591
No	66.2 ± 15.1	75.6 ± 13.8	9.5 (7.9 to 11.1), <0.001	
Yes	63.2 ± 14.1	72.6 ± 15.0	9.5 (5.8 to 13.1), <0.001	
Children				
Sex				0.616
Boys	55.7 (13.7)	69.6 (12.9)	14.0 (12.3 to 15.6), <0.001	
Girls	56.1 (13.2)	70.6 (13.2)	14.5 (12.8 to 16.2), <0.001	
Age				0.111
<9 years	54.4 (13.1)	69.1 (12.8)	14.7 (13.4 to 16.0), <0.001	
≥9 years	61.2 (13.5)	73.6 (13.2)	12.5 (9.9 to 15.1), <0.001	

Adjusted for cluster (city/school), sex, age, education, and household income.

**Table 5:** Subgroup analysis of changes in urinary sodium (mmol/24 h) by key characteristics.

Variable	Baseline (Mean ± SD)	Follow-Up (Mean ± SD)	Adjusted Diff (95% CI), *p*-Value	*p* Value for Heterogeneity Across Subgroups
Adults				
Sex				0.678
Male	167.3 (71.6)	158.7 (72.2)	−8.6 (−24.7 to 7.6), 0.298	
Female	144.1 (58.8)	138.4 (65.3)	−5.7 (−16.0 to 4.5), 0.270	
Age				
<35 years	152.9 (64.6)	146.3 (69.1)	−6.6 (−19.6 to 6.4), 0.318	0.796
35–<50 years	152.6 (63.7)	147.2 (68.2)	−5.4 (−19.3 to 8.5), 0.445	
≥50 years	149.1 (66.4)	136.9 (68.0)	−12.2 (−34.3 to 9.9), 0.276	
Relationship with children				
Parents	153.5 (64.4)	147.8 (69.3)	−5.7 (−15.3 to 4.0), 0.250	0.563
Grandparents	145.8 (64.5)	132.4 (62.8)	−13.4 (−34.3 to 7.5), 0.208	
Education status				0.422
Primary education or less	156.9 (56.9)	156.2 (67.7)	−0.7 (−22.6 to 21.2), 0.948	
Secondary education or High school education	153.4 (67.0)	148.1 (72.4)	−5.3 (−17.5 to 7.0), 0.397	
College education or above	147.4 (62.8)	133.7 (59.5)	−13.7 (−28.4 to 1.0), 0.068	
Family income per capita				0.192
<15,000	159.8 (69.7)	150.8 (66.1)	−8.9 (−25.3 to 7.5), 0.285	
15,000–30,000	145.9 (60.9)	147.3 (68.8)	1.3 (−13.4 to 16.1), 0.858	
>30,000	151.9 (62.8)	138.7 (70.0)	−13.2 (−27.5 to 1.1), 0.071	
Hypertension_overall				0.468
No	148.5 (60.9)	142.6 (65.4)	−5.9 (−14.9 to 3.1), 0.200	
Yes	174.6 (79.3)	160.7 (84.0)	−13.8 (−42.9 to 15.3), 0.348	
Children				
Sex				0.656
Boys	96.3 (39.8)	108.4 (70.9)	12.1 (1.9 to 22.4), 0.02106	
Girls	81.4 (33.8)	96.2 (42.9)	14.9 (7.6 to 22.2), <0.001	
Age				0.218
<9 years	87.9 (35.5)	103.9 (66.2)	16.1 (7.7 to 24.5), <0.001	
≥9 years	91.9 (42.1)	99.9 (42.5)	8.0 (−1.7 to 17.7), 0.106	

Note: The subgroup “Other family members” (*n* = 2) was excluded from the analysis due to insufficient sample size for meaningful statistical inference.

## Data Availability

The datasets generated and/or examined during the current study can be obtained from the corresponding author upon reasonable request.
